# Behavior, antioxidant, and metabolomics effects of *Allium tuncelianum*

**DOI:** 10.1002/fsn3.4022

**Published:** 2024-02-22

**Authors:** Nigar Abbak, Emirhan Nemutlu, Tuba Reçber, Asli San Dagli Gul, H. Turan Akkoyun, Mahire Bayramoglu Akkoyun, Gulderen Yilmaz, Suat Ekin, Ahmet Bakir, Okan Arihan

**Affiliations:** ^1^ Department of Physiology, Faculty of Medicine Hacettepe University Ankara Turkey; ^2^ Department of Analytical Chemistry, Faculty of Pharmacy Hacettepe University Ankara Turkey; ^3^ Department of Physiology, Veterinary Faculty Siirt University Siirt Turkey; ^4^ Department of Biochemistry, Veterinary Faculty Siirt University Siirt Turkey; ^5^ Department of Pharmaceutical Botany, Faculty of Pharmacy Ankara University Ankara Turkey; ^6^ Department of Biochemistry, Faculty of Science Van Yuzuncu Yil University Van Turkey

**Keywords:** *Allium*, *Allium tuncelianum*, antioxidant, anxiety, behavior, elevated plus maze, hotplate, metabolomics, rotarod, tunceli mountain garlic

## Abstract

Allium species are consumed extensively as folkloric medicine and dietary elements, but limited studies have been conducted on them. In this study, the effects of an ethanol–water extract obtained from the underground bulb of *Allium tuncelianum* (Kollmann) Özhatay, B. Mathew & Şiraneci (AT) on the behavioral, antioxidant, and metabolite parameters in rats were evaluated. AT was administered orally once a day at doses of 100 and 400 mg/kg to male Wistar albino rats for 10 consecutive days. The elevated plus maze, rotarod, and hotplate tests were used to examine anxiety‐like behaviors, locomotor activities, and pain perception in the rats, respectively. Additionally, untargeted metabolomic analyses were performed on plasma samples and AT extracts using two orthogonal analytical platforms. The phenolic components, mainly fumaric acid, malic acid, vanillic acid, quercetin‐3‐arabinoside, hydrocinnamic acid, and gallocatechin, were determined in the extract. In addition, arbutin, salicylic acid, trehalose, and nicotinic acid were analyzed in the extract for the first time. The AT extract did not decrease the catalase, glutathione peroxidase, or superoxide dismutase levels; however, diazepam decreased some of those parameters significantly in the brain, liver, and kidney. Although both the AT and diazepam treatments resulted in an increase in anxiolytic‐like effects compared to the control group, no significant differences were observed (*p* > .05). In the metabolomic analysis, significant changes were observed in the rats treated with AT and diazepam, and they caused significant changes in some metabolic pathways, including amino acid and fatty acid metabolism, compared to the control.

## Graphical abstract

Experimental design and procedure representing the model used in the study.

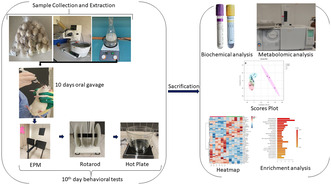

## 1 INTRODUCTION

Plants contain many chemical compounds that not only enable them to maintain their own physiological processes but also exhibit biological activities in other organisms that consume them. Polyphenols are important antioxidants that protect plants from the damage caused by oxidative stress induced by free radicals (Giglio et al., 2018).

One of the most effective and up‐to‐date methods used in identifying and determining the quantities of rich bioactive molecules and metabolites present in plants and other living organisms is metabolomics. Metabolomic research on complex sample types such as plant extracts and the plasma of mammals, gas chromatography–mass spectrometry (GC–MS), and liquid chromatography with high‐resolution mass spectrometry techniques are frequently used to increase metabolite coverage. GC–MS is one of the most effective techniques used in the identification and quantification of lipids, volatile metabolites, and even polar metabolites after derivatization (Gao et al., 2021; He & Aga, 2019). In non‐targeted metabolomic studies, liquid chromatography with high‐resolution mass spectrometry is an increasingly preferred technique due to its wider and higher sensitivity as a result of its ability to achieve the required level of mass accuracy for metabolite annotations (Gika et al., 2014; Vinaixa et al., 2016; Zeki et al., 2020). Although biological activities such as antioxidant, anxiolytic, locomotor, analgesic, learning, and memory have been studied in *Allium* species, studies concerning metabolomics following administration to experimental animals are insufficient for this genus.

Anxiety is one of the natural defense emotions that helps humans and many other species to survive (Steimer, 2002). However, when this emotion leads to an anxiety disorder, it can cause problems in an individual's physical, mental, and social life (Stein et al., 2017). Anxiety disorders are among the most common psychiatric disorders worldwide (Gustavsson et al., 2011), and antidepressants are often used in their treatment. Benzodiazepines make up a large portion of the antidepressants used to treat anxiety (Stahl, 2002). However, due to their side effects, their use is limited (Bandelow et al., 2017). Therefore, in addition to the primary treatment methods, there is a need for safer drugs and natural products with fewer side effects for the treatment of anxiety disorders (Lowe et al., 2022).

Species of the genus *Allium* in the human diet are one of the primary sources of flavonoids, phenols, and flavonols, which belong to the polyphenol group (Sasi et al., 2021). Various *Allium* species are used worldwide due to their medicinal properties, including hypoglycemic, hypolipidemic, memory‐enhancing, antioxidant, anxiolytic, antidepressant, anesthetic, wound‐healing, and anti‐inflammatory effects (Ahmed et al., 2021; El‐Saber Batiha et al., 2020). Both phytochemical and biological research on *Allium* species are increasing; however, the lack of in vivo and metabolomics studies has resulted in an insufficient understanding of the biochemical mechanisms that occur following *Allium* species.

Türkiye is quite rich in *Allium* species and has many endemic species. In a study conducted by Rocchetti et al., methanol–water extracts of wild *Allium* species grown in Türkiye, including *A. scabriflorum*, *A. atroviolaceum*, *A. panikulatum*, *A. vineale*, *A. goekyigitii*, *A. isauricum*, and *A. trachycoleum*, were investigated. The phytochemical profiles and in vitro antioxidant activities, as well as the inhibitory properties on acetylcholinesterase, butyrylcholinesterase, α‐amylase, α‐glucosidase, and tyrosinase, were examined. The study emphasized that the main class of bioactive compounds identified from *Allium* species was polyphenols. In general, anthocyanins, flavanols, and other flavonoids, which are phenolic compounds, have been identified. The most represented compounds in flavonoids are peonidin 3‐O‐rutinoside, delphinidin 3,5‐O‐diglucoside, and cyanidin 3‐O‐glucosyl‐rutinoside (among anthocyanins), and catechin/epicatechin, hesperidin, chrysoeriol 7‐O‐glucoside, and quercetin 3‐O‐rutinoside, and other flavonoids (Rocchetti et al., 2021). In a study by Izol et al., the phytochemical profiles of 12 *Allium* species collected from the eastern Anatolian region of Türkiye were examined, and quinic acid, malic acid, vanillic, and p‐coumaric acid were identified as common phenolic compounds in the ethanol extracts of the plants' aerial parts (Izol et al., 2021).

Takim et al. examined the phenolic quantity and content in AT extracts using different solvents. Among these phenolic chemicals, malic acid, kainic acid, cinnamic acid, vanillic acid, and fumaric acid, as well as catechin and quinic acid, the major phenolic components of AT, were found in different articles, suggesting antioxidant properties for this plant species (Takim et al., 2021; Takim & Kutlu, 2020).

*Allium tuncelianum* (Kollmann) Özhatay, B. Matthew & Şiraneci (mountain garlic) (AT) is an endemic *Allium* genus species in Türkiye. AT is widely used in this local region for culinary and ethnobotanical reasons. Still, the scientific literature on behavior and locomotor activity, as well as its metabolomic effects on laboratory animals following the administration of *Allium* extracts, is insufficient.

The study aimed to evaluate AT plant extract's anxiolytic‐like effects, analgesic, and antioxidant activity in rats (100 and 400 mg/kg, orally administered for 10 days) through behavioral, biochemical, and metabolomic parameters. In this study, the antioxidant activity in the liver, kidney, and brain tissue, anxiolytic‐like effects, analgesic activity, locomotor activity, and alterations in the plasma metabolite profiles were investigated.

## 2 MATERIALS AND METHODS

### 2.1 Animals

Male Wistar albino rats 4–8 weeks old (200–300 g) were housed in appropriately sized plexiglass cages at the Department of Physiology, Faculty of Medicine, under constant temperature (22 ± 2°C) and a 12 h light/dark cycle. Standard rat food (pellets) and tap water were provided ad libitum. After acclimatization to the environment, 24 animals were used, consisting of a negative control group (administered 10 mL/kg of distilled water), AT100 and AT400 groups (given 100 and 400 mg/kg, respectively), and a diazepam group (administered 1.5 mg/kg of diazepam), with each group containing 6 animals (*n* = 6). The AT ethanol–water extract and diazepam were dissolved in distilled water and administered orally at a volume of 10 mL/kg for 10 consecutive days at the same time every day. Doses were selected according to studies in the literature (Atila et al., 2019; Takım, 2015). Behavioral tests were conducted 1 h after the last treatment on day 10 (Akindele et al., 2012). The elevated plus maze (EPM), rotarod, and hotplate tests were used, respectively.

All of the animal behavior experiments were recorded using a camera mounted on the ceiling that was connected to a computer. At the end of the tests, the rats were euthanized under anesthesia with ketamine (90 mg/kg, intraperitoneally (i.p.)) and xylazine (10 mg/kg, i.p.).

### 2.2 Preparation of the plant extracts

*Allium tuncelianum* (Mountain Garlic) is a naturally distributed (and endemic) species in Tunceli (Türkiye) province. The species identification of *A. tuncelianum* samples purchased from local sellers in Tunceli (Türkiye) in August 2022 was carried out and performed by Assoc. Prof. Dr. Gulderen Yilmaz. After the plant material was cut into small pieces and weighed (120 g), it was extracted with an ethanol–water mixture (4:6, v/v) at room temperature by maceration for 3 days and 8 h. Ethanol was removed from the total extract using a rotavapor. The remaining total extract was lyophilized. Then, 40 g of the total dry extract was obtained from 120 g of plant sample. The yield was calculated as 33%, and the resulting dry plant extract was stored in a refrigerator at 4°C until used in the study.

### 2.3 Elevated plus maze (EPM)

The EPM test is one of the most commonly used methods to evaluate anxiety‐like behavior in rats. The test consists of two open and two closed arms that intersect each other in a plus shape, located 50 cm above the floor. Rats prefer to stay in their closed arms due to their fear of open spaces (Walf & Frye, 2007). After treatment, each rat was placed in the center of the EPM, facing an open arm. The following observations of the group were made during the 5 in test: the number of entries into the open and closed arms, the time spent in the open and closed arms, the percentage of entries into the open arms, and the percentage of time spent in the open arms (Emamghoreishi et al., 2005).

### 2.4 Rotarod

The rotarod test was used to evaluate the motor coordination of the rats (Monville et al., 2006). First, an adaptation test was performed for 2 min on a rotating rod that moved at 5 rpm, and the rats that fell off were placed back on the rod. After the adaptation test, the time that the rats could stay on the rotating rod moving at 15 rpm was recorded as the latency (Ferrante et al., 2002).

### 2.5 Hotplate test

The hotplate test was used to evaluate pain response in the assessment of neurological complications. In this test, a 52°C heat stimulus was applied to the hind paw of the rat, and the latency to withdraw or tremor was recorded (Deuis et al., 2017).

### 2.6 Oxidative stress parameters

Brain, kidney, and liver tissues were extracted bilaterally from the rats for the evaluation of oxidative stress parameters; catalase (CAT), glutathione peroxidase (GSH‐Px), and superoxide dismutase (SOD) antioxidant enzymes. The tissues collected for evaluation of the oxidative stress parameters were washed with phosphate‐buffered saline and stored in a deep freezer (−80°C) until the biochemical analysis.

### 2.7 Liver, kidney, and brain tissue preparation

Liver, kidney, and brain tissues were extracted for the evaluation of oxidative stress parameters, such as CAT, GSH‐Px, and SOD antioxidant enzymes, in the rats. The tissue samples were weighed at around 500 mg on an analytical scale in a tube. Then, cold Tris buffer (1 mmol/L of ethylenediaminetetraacetic acid, 0.32 mol/L of sucrose, and 10 nmol/L of Tris‐hydrochloride, pH 7.4) was added to the tube in an amount 10 times the weight of the tissue. The samples were homogenized using a homogenizer (Ultra Turrax T25, IKA, Staufen, Germany) and stored at −20°C in a refrigerator. After vortexing, the samples were transferred into porcelain crucibles and subjected to ultrasonic treatment at a frequency of 20 KHz in an ultrasonic disruptor to break the cell membrane, followed by centrifugation at 1600 rpm for 30 min (Xia et al., 1995). The clear supernatants were transferred into Eppendorf tubes. All of the procedures were performed at 4°C.

### 2.8 Determination of the antioxidant enzyme activities and total protein concentration

Antioxidant enzyme activities in the liver, kidney, and brain tissues were determined spectrophotometrically. The SOD (EC 1.15.1.1) activity was determined according to the method of Sun et al. (Sun et al., 1988). The tissue enzyme activity of the GSH‐Px (EC 1.11.1.9) was determined using the method of (Paglia & Valentine, 1967), while the CAT (EC 1.11.1.6) activity was determined according to the method of (Aebi, 1984). The total protein concentrations of the liver, kidney, and brain tissue homogenates were measured spectrophotometrically (Lowry et al., 1951) using bovine serum albumin solution as the standard solution.

### 2.9 Metabolomic analyses

The metabolomic analysis was performed using two orthogonal analytical platforms, including GC–MS and LC‐qTOF‐MS. The methods were adopted from our previous studies, and the method parameters were briefly given therein (Eylem et al., 2020; Gonulalan et al., 2020; Kart et al., 2020).

#### 2.9.1 Sample preparation for the metabolic analysis

First, 1 mL of blood was drawn from the rats' *vena cava inferior* into EDTA tubes. After centrifugation of the blood samples at 5000 rpm for 10 min, the plasma samples were stored at −80°C until analysis. For the sample preparation, 100 μL of the plasma samples were extracted using a 900 μL methanol:water mixture (9:1, v/v). After gently vortexing for 1 min and centrifugation at 15,000 rpm for 5 min, two separate 400 μL aliquots were evaporated to dryness for the GC–MS and LC‐qTOF‐MS analyses.

In the GC–MS study, the dried samples were derivatized with 20 μL of methoxyamine hydrochloride (20 mg/mL in pyridine) at 30°C for 90 min and 50 μL of N‐methyl‐N‐(trimethylsilyl)trifluoroacetamide +1% trimethylchlorosilane at 37°C for 30 min. The derivatized samples were transferred into vials and analyzed using GC–MS.

For the LC‐qTOF‐MS analyses, the dried‐out samples were reconstituted with 200 μL of acetonitrile/water (1:1, v/v) containing 0.1% of formic acid, vortexed for 1 min, and centrifuged at 15,000 rpm for 10 min. Then, 180 μL of the supernatant was transferred into a vial and analyzed by LC‐qTOF‐MS.

#### 2.9.2 GC–MS‐based metabolomic profile analyses

GC–MS analyses were performed using a Shimadzu GCMS‐QP2010 Ultra GC–MS (Shimadzu Corporation, Kyoto, Japan) with a dose‐base (5%‐phenyl)‐methylpolysiloxane (DB‐5MS) stationary phase column (30 + 10 m DuraGuard × 0.25 mm i.d. and 0.25 μm film thickness). The oven temperature was initially held at 60°C for 1 min. Afterwards, the temperature was raised with a gradient of 10°C/min until 32°C. This temperature was held for 10 min before cooling down. The MSD transfer line temperature was set to 290°C. The solvent delay was set for 5.90 min. The flow through the column was held constant at 1 mL He/min. The mass range was 50–650 Da. The injection volume was 1 μL. The run time was 37.5 min.

#### 2.9.3 LC‐qTOF‐MS‐based metabolomic profile analyses

The metabolite analysis was performed using a C18 column (2.1 × 100 mm, 2.1 μm) with an Agilent 6530 LC‐qTOF‐MS system (Agilent Technologies, Santa Clara, CA, USA) in both negative and positive ionization modes. The mobile phase included solvent A (0.1% formic acid) and solvent B (acetonitrile with 0.1% formic acid) with gradient elution (0–1 min, 90% B, 1–10 min 90%–10% B, 10–14 min 10% B, 14–15 min 10%–90% B, and 15–25 min 90% B). The flow rate was 0.3 mL/min. The injection volume was 10 μL. The analyses were performed at the electrospray ionization source with the following parameters: Capillary voltage of 4000 V and capillary temperature of 300°C. The auto‐tandem MS/MS spectra of the metabolites were recorded between 100 and 1700 m/z above the 200‐count threshold. To identify the peaks in the resulting data matrix, MS/MS spectra obtained by applying different collision energies (10, 20, and 40 eV) to the quality control samples (created by pooling the samples) were scanned against libraries.

#### 2.9.4 Interpretation of profiles

The complex chromatograms were deconvoluted using data‐independent acquisition liquid chromatography‐coupled MS (MS‐DIAL) software, and the peak retention times were adjusted to create data matrices. The peaks of the resulting metabolites were identified using Fiehn retention index libraries for the GC–MS analysis and MS/MS data for the LC‐qTOF‐MS analysis. The merged data from the GC–MS and LC‐qTOF‐MS analyses were evaluated using multivariate statistical techniques, including principal component analysis (PCA) and partial least squares discriminant analysis (PLS‐DA), to identify any systemic errors and the important metabolites in separating the groups, respectively. The metabolites that caused differentiation in the PLS‐DA analysis were obtained using variables important in the project (VIP) plots. The metabolite with the highest VIP value was the one that showed the most statistical difference between the groups and was considered a biomarker. Finally, enrichment analysis was performed using the obtained metabolites, and the most altered pathway in the phenotype was identified.

### 2.10 Statistical analysis

Statistical analyses were performed in IBM SPSS Statistics for Windows 23.0 (IBM Corp., Armonk, NY, USA). Variance analyses and related post hoc tests were used in the behavioral, antioxidant, and metabolomic analyses.

## 3 RESULTS

### 3.1 Behavioral data

#### 3.1.1 Effect of AT on the anxiety‐related behaviors

After the rats were administered AT, there were non‐significant increases in the time spent in the open arms, the number of entries into the open arms, the time spent in the open arms in the AT100 and AT400 groups when compared to the control (Table 1).

**TABLE 1:** Different doses of AT ethanol–water extract effects on the EPM, hotplate, and rotarod tests at *p* < .05. Groups: Control, AT100 (100 mg/kg), AT400 (400 mg/kg), and diazepam (1.5 mg/kg).

	Control (10 mL/kg water)	AT100 (100 mg/kg)	AT400 (400 mg/kg)	Diazepam (1.5 mg/kg)
Number of entries into the open arms	1.8 ± 1.3	2.6 ± 1.6	2.1 ± 1.7	2.5 ± 1.8
Percentage of entries into the open arms	16.9 ± 13.9	30.2 ± 12.3	30.1 ± 23.0	28.4 ± 20.1
Time spent (s) in the open arms	19.4 ± 16.9	25.3 ± 16.8	22.1 ± 18.5	28.1 ± 20.1
Percentage of time spent in the open arms	9.7 ± 8.4	12.5 ± 8.9	10.4 ± 9.9	15.2 ± 15.2
Endurance time on the rotarod	77.6 ± 23.7	118.3 ± 34.2	117.2 ± 63.5	136.2 ± 32.1
Latency to withdraw on the hotplate	4.8 ± 0.9	5.3 ± 1.8	4.7 ± 0.9	4.5 ± 1.2

#### 3.1.2 Antioxidant results

The antioxidant enzyme (CAT, SOD, and GSH‐Px) activities in the liver, kidney, and brain tissue samples are presented in Table 2. When the liver tissues were examined, the diazepam group had significantly lower CAT enzyme activity (*p <* .01) and GSH‐Px (*p* < .05) than the control group. The diazepam group also had significantly lower CAT activity compared to the AT400 group (*p* < .05). When the kidney tissues were examined, the diazepam group had significantly lower CAT enzyme activity (*p <* .001) than the control group as well as AT400 group (*p* < .05). The diazepam group also had significantly lower CAT activity in the kidney compared to AT400 (*p* < .05). When the brain tissues were examined, the diazepam group had significantly lower GSH‐Px enzyme activity (*p* < .05) than the control group (Table 2).

**TABLE 2:** Mean antioxidant enzyme (CAT, SOD, and GSH‐Px) activities of the liver, kidney, and brain tissue samples of the control, AT100 (100 mg/kg), AT400 (400 mg/kg), and diazepam (1.5 mg/kg) groups.

	Parameters	Control (10 mL/kg water)	AT 100 (100 mg/kg)	AT 400 (400 mg/kg)	Diazepam (1.5 mg/kg)
Liver	CAT (EU/mg protein)	8.30 ± 1.29^**b**^	6.82 ± 1.18	7.67 ± 1.39^c^	5.68 ± 1.08^**b.c**^
	SOD (EU/mg protein)	832.88 ± 98.04	740.54 ± 54.36	820.79 ± 102.46	711.47 ± 31.31
	GSH‐Px (EU/mg protein)	1.79 ± 0.17^**c**^	1.65 ± 0.23	1.70 ± 0.29	1.46 ± 0.08^**c**^
Kidney	CAT (EU/mg protein)	3.82 ± 0.31^**a**^	3.23 ± 0.69	3.42 ± 0.44^**c**^	2.38 ± 0.64^**a.c**^
	SOD (EU/mg protein)	826.85 ± 75.62	776.05 ± 47.55	792.56 ± 28.21	739.15 ± 61.91
	GSH‐Px (EU/mg protein)	1.74 ± 0.20^**b**^	1.52 ± 0.13	1.61 ± 0.09	1.42 ± 0.08^**b**^
Brain	CAT (EU/mg protein)	3.58 ± 0.91	3.02 ± 0.83	3.36 ± 0.50	2.63 ± 0.49
	SOD (EU/mg protein)	1591.31 ± 216.22	1555.34 ± 140.71	1587.66 ± 427.62	1506.71 ± 203.96
	GSH‐Px (EU/mg protein)	0.46 ± 0.13^**c**^	0.32 ± 0.053	0.37 ± 0.10	0.29 ± 0.06^**c**^

Abbreviations: CAT, Parameters: catalase; GSH‐Px, glutathione peroxidase; SOD, superoxide dismutase.

a: *p* < .001, b: *p* < .01. c: *p* < .05 (same letters indicate significant differences between the groups). ± values represent the standard error of the means. a, b, and c indicate significance at *p* < .001, *p* < .01, and *p* < .05, respectively. CAT.

#### 3.1.3 Metabolomics results

**FIGURE 1:**
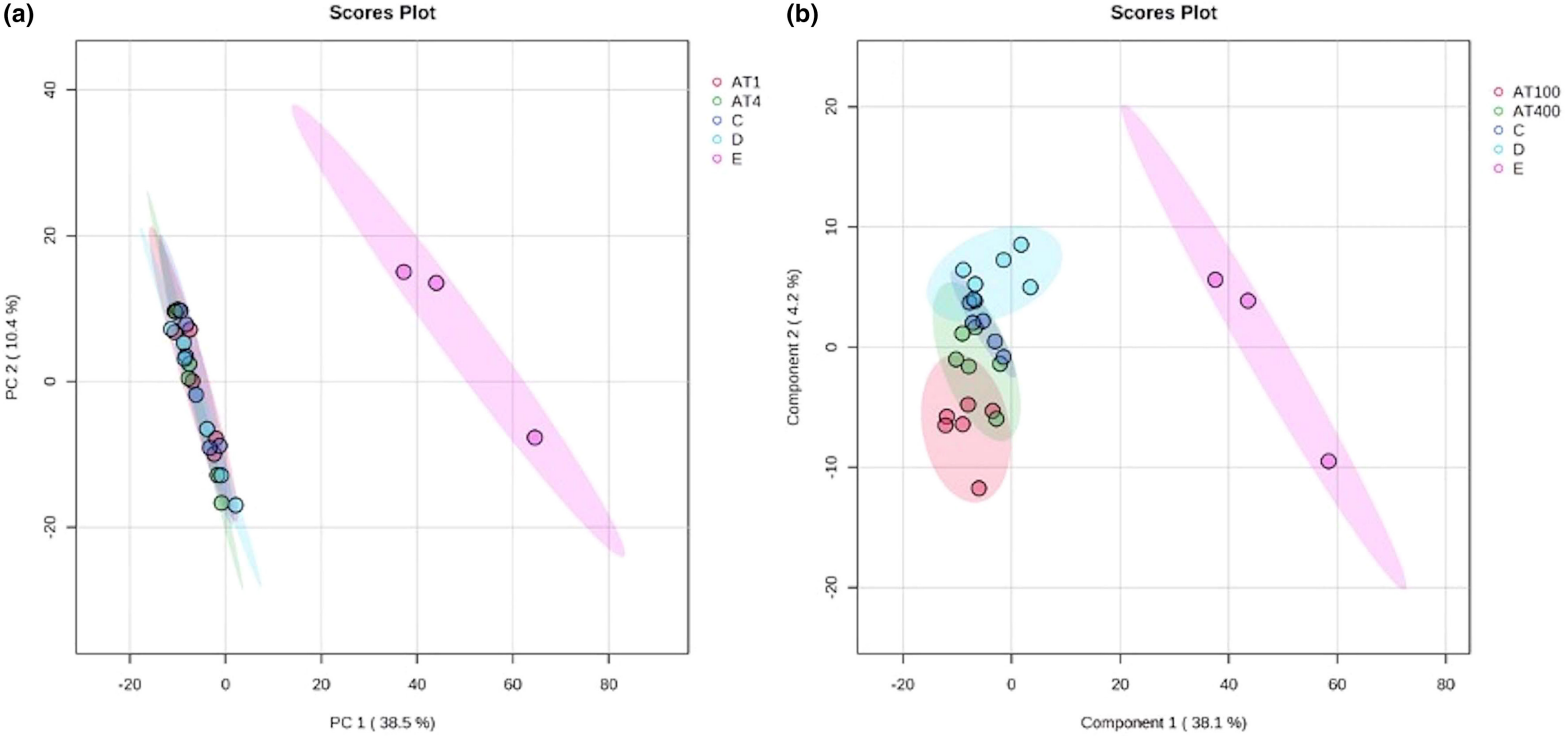
(a) PCA and (b) PLS‐DA Score plots for metabolite levels in plasma samples from Control, Diazepam, AT 100, and AT 400 groups.

**FIGURE 2:**
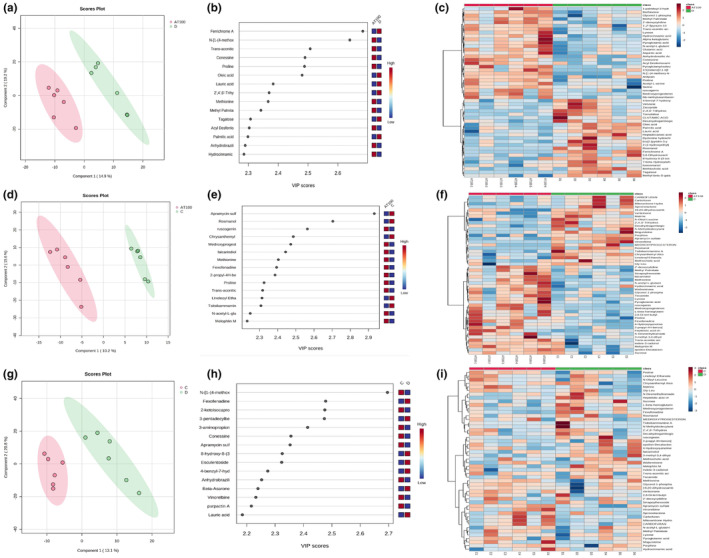
Comparison of the plasma metabolic profiles of the groups: (a) PLS‐DA score plot of AT100 vs Diazepam groups (b) VIP charts of metabolites that are effective in separating AT100 and Diazepam groups, (c) Heat map for metabolites in AT100 and Diazepam groups (d) PLS‐DA score plot of AT100 vs Control groups (E) VIP charts of metabolites that are effective in separating AT100 and Control groups, (f) Heat map for metabolites in AT100 and Control groups, (g) PLS‐DA score plot of Diazepam vs Contol groups (h) VIP charts of metabolites that are effective in separating Diazepam and Control groups, (i) Heat map for metabolites in Diazepam and Control groups. PCA, Principal component analysis; PLS‐DA, Partial least squares discriminant analysis; VIP, Variables important in the project.

After the metabolomic analyses, a total of 114 primary and 1068 secondary metabolites were identified. The merged data from the GC–MS and LC‐qTOF‐MS analyses were evaluated using PCA and PLS‐DA (Figure 1). The PCA analysis indicated that there were no systemic errors or outliers in the dataset. Subsequently, pairwise comparisons with the PLS‐DA analysis showed that the groups had different metabolomic profiles (Figure 2a,d,g). The VIP plots showed significantly altered metabolites that caused differentiation in the PLS‐DA analysis (Figure 2b,e,h). During the group comparisons, only the AT100 and control groups were selected since the main aim was to investigate the effect of the given plant extract on rat metabolites. The data of the AT400 group showed a very high similarity with the control group in the PCA plot (Figure 1a,b), so the comparison was made between the AT100, diazepam, and control groups, which showed the actual difference (Figures 2c,f,i).

According to HeatMap, methionine, glycerol 1‐phosphate, methyl palmitate, 1′,2′‐f pyrazine, hydrocinnamic acid, alpha ketoglutaric acid, glutamic acid, aspartic acid, ruscogenin, medroxyprogesterone, nb‐methylusambarensine, and proline levels were higher at the AT100 group compared to the diazepam group, while the oleic acid, palmitic acid, lauric acid, glutamic acid, methiocholic acid, 8‐hydroxy‐8‐(3‐octyloxiran‐2‐yl)octanoic acid, and rosmanol levels were higher at the diazepam group (Figure 2c).

When a similar comparison was made between the AT100 and control groups, it was found that the AT100 group had higher levels of methyl palmitate, hydrocinnamic acid, ruscogenin, proline, pyroglutamic acid, 2,6‐di‐tert‐butyl‐1,4‐benzoquinone, and fexofenadine levels, while carbofuran, mitoxantrone hydrochloride, apramycin sulfate, vinorelbine, and rosmanol levels were higher in the control group (Figure 2f).

According to the pairwise comparisons, the 2‐amino‐1‐phenylethanol, 2‐deoxycytidine, beta‐alanine, citric acid, methionine, methyl palmitate, methyl stearate, myoinositol, N‐acetyl‐L‐glutamic acid, phenylalanine, proline, pyroglutamic acid, threitol, trans‐aconitic acid, and valine levels were significantly higher in the AT100 group compared to the control group, while the trehalose level was lower.

In regard to the secondary metabolites, the following compounds were found at significantly higher levels in the AT100 group compared to the control group: (2E,4E)‐N‐(2‐methylpropyl)deca‐2,4‐dienamide, 1,4‐androstadiene‐3, 17‐dione, 4‐ydroxybenzoylcholine, 4‐hydroxyquinoline, andrastin D, baquiloprim, FA 18:3 + 1O, and falcarindiol. Secondary metabolites austrobuxusin F, biliverdin F, decahydrogambogic acid, dehydroabietamide, isosafrole, and tsitsikammamine A were found at significantly lower levels in the AT100 group compared to the control group.

**FIGURE 3:**
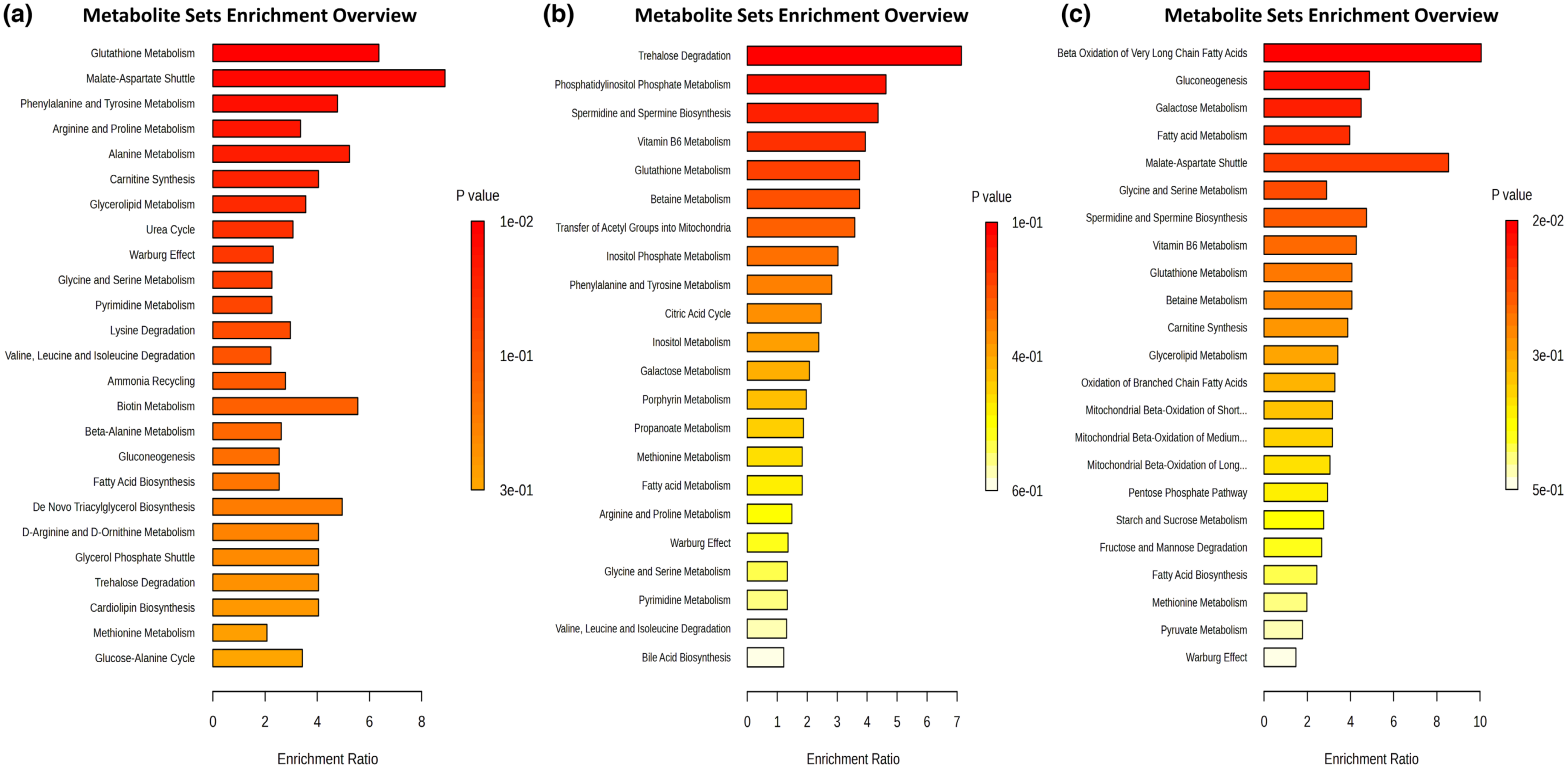
Enrichment pathway derived from enriching pathway analysis of statistically significant altered metabolites. (a) Diazepam vs AT100, (b) AT100 vs Control, and (c) Diazepam vs Control.

Enrichment analyses were conducted for each group to identify the altered metabolomic pathways using significantly changed metabolites (*p <* .05) between the paired groups (Figure 3a–c). In the comparison of AT100 and diazepam groups, there were statistically significant changes in the glutathione metabolism, malate–aspartate shuttle, phenylalanine and tyrosine metabolism, and arginine and proline metabolism; in the AT100 and control groups, alterations were observed in the trehalose degradation, phosphatidylinositol phosphate metabolism, and spermidine biosynthesis; and between the diazepam and control groups, statistical significance was found in the beta‐oxidation of the very long‐chain fatty acids, gluconeogenesis, galactose metabolism, malate–aspartate shuttle, and glycine serine metabolism (Figure 3a–c).

#### 3.1.4 Plant extract content

The chemical composition of the ethanol–water AT extract was studied using two orthogonal analytical methods. The main phenolic components detected in the plant extract were fumaric acid, malic acid, valine, quercetin‐3‐arabinoside, hydrocinnamic acid, and gallocatechin. In addition, arbutin and salicylic acid, as well as trehalose and nicotinic acid metabolites, were detected.

The most abundant molecules in the plant extract were eupomatenoid 5, 4′,7‐di‐O‐methylnaringenin, kakkalide, luteolin C‐glucoside C‐xyloside, 6,3′‐imethoxyflavone, and 4′,7‐di‐O‐methylnaringenin from the flavonoids, cuminyl alcohol from the monoterpenoids, 9,13‐epoxy‐3,15,16,18‐labdanetetrol from the diterpenoids, and tigogenin from the triterpenoids.

Among some of the groups of fatty acid compounds, the most abundant were azelate and azelaic acid in the medium‐chain fatty acids, (Z)‐9,12,13‐trihydroxyoctadec‐15‐enoic acid and stearic acid in the long‐chain fatty acids, and tetracosanoic acid in the very long‐chain fatty acids. Coumarin and its derivatives, including coumarin and excavatin L, were more abundant than the other coumarin derivatives. The most abundant molecule from the linoleic acid and its derivatives was linoleic acid. The most abundant amino acids among the different types were pyroglutamic acid, L‐beta‐homovaline, L‐lysine, and L‐ornithine.

## 4 DISCUSSION

This study, in which AT was administered to rats at doses of 100 and 400 mg/kg, focused on its behavioral, antioxidant, and metabolomic effects, as well as comparisons with the control and diazepam groups. The results showed prominent changes in the metabolomic content as a result of the AT, at a dose of 100 mg/kg, or diazepam and antioxidant parameters. In addition, some insignificant alterations were observed in the behavioral parameters.

### 4.1 Behavior tests

The current study results suggest that the changes observed in the EPM may indicate the anxiolytic‐like effect of AT. Although no significant increase was observed, the number and percentage of entries into the open arms, as well as the time and percentage spent in the open arms, increased in the groups treated with AT. This difference may be more significant in further studies with a larger number of subjects. A similar pattern was also observed in the diazepam group, as expected. In a study on anxiety and depression‐like behaviors in rats related to *Allium*, a methanol extract of *Allium cepa* Linn was administered to rats for 7 days at doses of 200 and 400 mg/kg, and behavioral tests were performed. The EPM, open‐field test (OFT), and light–dark box test showed that the methanol extract had an anxiolytic‐like effect at 400 mg/kg that was comparable to that of the standard drug diazepam (Pitchaiah et al., 2015). Although the doses and duration used in the mentioned article were similar to those in the current study, the effect was not similar. This may have been due to the use of two different *Allium* species and the use of a methanol extract in the aforementioned study compared to an ethanol–water extract in the current study.

In another study, an aqueous extract of *Allium sativum* (AS) was administered via gavage at doses of 100, 250, and 500 mg/kg for 10 days to rats with streptozotocin (STZ)‐induced diabetes. Anxiety tests were performed using EPM as well as OFT, and depression tests were also performed. Significant decreases were observed in the anxiety and depression‐like behaviors in the diabetic rats with a dose of 500 mg/kg of AS. It was emphasized that AS may reduce potential brain oxidative stress in diabetic rats and also reduce anxiety and depression‐related behaviors (Rahmani et al., 2020). However, it is important to note that this antioxidant effect was assessed only in AS‐administered diabetic rats. No lone AS group was assessed in the study. The difference in the *Allium* species, extract used in their study, and experimental protocol may have been the reason for the different results obtained compared to the current study, both chemically and behaviorally.

Although an increase in anxiolytic‐like behaviors was observed in the diazepam and AT groups in the current study, the lack of a clear anxiolytic effect may have been due to the low anxiolytic effect of AT, the individual characteristics of the animals, and the insufficient number of animals studied. Although the results of unconditional anxiety tests are gathered quickly, they may sometimes be insufficient to create conflict or stress in animals. Thus, further studies with a conditional test on AT may give clearer results.

The rotarod test results showed no significant difference in the walking period on the rotarod between the control and treatment groups, indicating that the administrations performed did not alter locomotor activity. There are no previous studies on the behavior of the AT, which makes this current study the first to provide data to the literature. Further research on the broad perspective of behavior studies will provide a better understanding of the physiological and behavioral effects of this species.

### 4.2 Analgesic effects

The prolonged use of traditional analgesics to manage pain is associated with toxicity, which highlights the need for new analgesics (Elvir‐Lazo et al., 2021). The central analgesic effect of different *Allium* garlic species was evaluated using the hotplate test, which is one of the experimental pain methods (Dange et al., 2016). Studies have reported the antinociceptive effects of *Allium* species such as *A. ampeloprasum* (Abbas, 2019) and *A*. *jesdianum* (Khaksarian et al., 2008). In studies using the hotplate test, it has been shown that different *Allium* species generally delay the response time to pain compared to the control group in animals. The analgesic effect of AT has not been studied before. Although an increase in the withdrawal latency from the hotplate test has been observed to indicate an antinociceptive effect, statistical significance was not reached. An increase in the number of animals in each group, as well as the dose and duration of the administration of the substance, may augment the possible analgesic effect in future studies.

### 4.3 Antioxidant parameters

Since *Allium* species are important food components in the human diet, numerous biological activity and antioxidant studies have been conducted on them. Studies comparing AT and AS extracts obtained in different solvents have shown that AT has a higher antioxidant capacity than the well‐known AS (Şehitoglu et al., 2018). In an in vitro study by Karaaslan et al., the antioxidant activity of AT extract was investigated using acidified water, acetonitrile, methanol, and ethanol. It was emphasized that the AT methanol extract was more effective than other extracts in scavenging 2,2′‐azino‐bis (3‐ethylbenzothiazoline‐6‐sulfonic acid) radical scavenging capacities and cupric‐reducing antioxidant capacity radicals (Karaaslan et al., 2019). Similarly, Karakavuk et al. found that AT methanol extract had 2,2′‐azino‐bis (3‐ethylbenzothiazoline‐6‐sulfonic acid), cupric‐reducing antioxidant capacity, and free radical removal capacity (2,2‐diphenyl‐1‐picrylhydrazyl) inhibition compared to aqueous and ethanol extracts (Karakavuk, 2021).

Only two studies investigated the antioxidant effects of AT in vivo in pathological models. Takim et al. administered AT ethanol extract orally to rats at doses of 250 and 500 mg/kg/day for 30 days, along with intraperitoneal 7,12‐dimethylbenz(a)anthracene, a toxic cancerogenic substance. The protective effects of AT on the CAT enzyme activity in the kidney, SOD activity in the intestine and liver, GSHx enzyme activity in the liver, and MDA enzyme in the kidney were statistically significant compared to the 7,12‐dimethylbenz(a)anthracene‐treated group (Takim, 2015). In addition, Atila et al. administered a single dose of 50 mg/kg STZ and daily oral doses of 250 mg/kg oral AT to rats for 28 days. They reported that the SOD and CAT activities in the kidney and liver significantly increased in the diabetic models treated with AT (Atila et al., 2019). In both studies, the antioxidant effect was observed in the presence of a pathology; therefore, it can be considered that it has a protective effect in oxidative stress conditions.

According to the results of the current study, diazepam caused a significant decrease in the antioxidant enzyme levels compared to the control group in the liver (CAT, GsH‐Px), kidney (CAT, GsH‐Px), and brain (GsH‐Px) of the rats. The obtained antioxidant enzyme results indicate that diazepam causes oxidative stress, while AT extracts do not have such an effect.

Different studies showed that *Allium* extracts alleviate decreased antioxidant levels and reduce the physiological impact of pathologies when administered against different pathological conditions or metabolic problems such as STZ‐induced diabetes (Atila et al., 2019; Ayodhya et al., 2010; Takim et al., 2021). The antioxidant effects of lone *Allium* extracts were not assessed in those studies. In the current study, the *Allium* extracts may not have exhibited a significant effect on antioxidant levels since they were administered to animals without any obvious pathology or metabolic problems. In addition, the administration period of 10 days may have been a factor in the lack of a significant antioxidant effect. The two studies aforementioned above, which showed significant results, administered AT orally for 28 and 30 days, respectively.

Although various studies have shown that the consumption of *Allium* species is beneficial for human health due to their biological effects, particularly their antioxidant properties, some studies have suggested the harmful effects of onion consumption in certain mammals. The sulfur compounds found in *Allium* may cause toxicological effects such as methemoglobinemia and Heinz body formation in the erythrocytes (Salgado et al., 2011). The toxicity of *Allium* depends on the amount and frequency of consumption, and it has been shown to cause Heinz body formation in the erythrocytes in many animals (Lee et al., 2006; Salgado et al., 2011). Depending on the studied species of *Allium*, pathological effects have been observed in most animals due to their chemical content and biological activity. In the current study, the significant changes in bilirubin, a breakdown product of hemoglobin in the erythrocytes, as a result of the AT administration draw attention to this point. Although this does not indicate a direct toxic effect, further studies are needed to investigate the detailed biological effects of this species.

### 4.4 Metabolomics

The studies in the literature have focused on endogenous metabolites of the AT. No studies have investigated the changes in metabolites in the tissues and biological materials of animals after administering the AT extract at different doses. The plasma samples of the animal groups and plant extract metabolomic profiles were analyzed using GC–MS and LC‐qTOF‐MS. The data were first evaluated using PCA analysis and no significant systemic errors or outliers were found in the dataset (Figure 1a).

The PLS‐DA analysis was used to obtain more detailed information about the data by superimposing the data groups from each other (control, AT100, AT400, and diazepam). The PLS‐DA score plot clearly separated the control, AT100, and diazepam groups (Figure 1b). However, since the AT400 group was not entirely separated from the AT100 and diazepam groups, it was not included in the metabolomic analysis (Figure 1b). Data obtained from the AT 100 group in the in vivo study were deemed more suitable for interpretation in the metabolomic analysis. In the pairwise comparison of the groups, the PLS‐DA score plots showed a significant separation trend in the rat metabolism due to AT treatment compared to the control group as well as the diazepam group (Figure 2a,d,g). In addition, enrichment analyses were conducted for each group to identify altered metabolomic pathways (Figure 3). These analyses indicated significant changes in the amino acid and lipid metabolism associated with the administration of AT at a dose of 100 mg/kg. Since this plant is an important local food source, these data are valuable for different aspects of public health. The metabolomics analysis results showed that the administration of AT led to significant changes in the metabolic profiles, without causing any changes in regard to oxidative stress or behavior. On the other hand, the administered diazepam induced significant oxidative stress as well as potent metabolic alterations.

The metabolites that caused differentiation in the PLS‐DA analysis were obtained using VIP plots (Figure 2b,e,h). The metabolite with the highest VIP value was the one that showed the most statistical difference between the groups and was considered a biomarker. Although important metabolites were identified from the VIP plots, the changes in the concentrations of which metabolites increased or decreased within the groups were determined by regression analysis coefficients. When AT100 was compared with diazepam and control, such metabolites commonly increased in the AT100 group were methionine, methyl palmitate, hydrocinnamic acid, proline, N‐acetyl‐glutamic acid, ruscogenin, glycerol‐1‐phosphate, lyxose, and pyroglutamic acid. In addition, pathway analysis was performed using metabolites, and the most altered pathway in the phenotype was identified.

The findings of the metabolite analysis demonstrated that both AT and diazepam altered the primary and secondary metabolite profiles in the rats. According to enrichment analysis, the augmentation in the beta‐oxidation pathway, gluconeogenesis, and fatty acid metabolism suggests that lipid metabolism and gluconeogenesis were significantly increased in diazepam‐administered rats (Figure 3c). The oxidation of branched‐chain fatty acids and mitochondrial beta‐oxidation of short‐medium‐long fatty acids remind us of an augmentation of lipid metabolism. In accordance with our data, Alvarez‐Mora et al. investigated metabolite changes during diazepam and irbesartan administration in glass eels. Dysregulation in the levels of twelve lipids, most of which have functions related to energy and structural functions, was observed. It has been emphasized that this situation may be related to oxidative stress, inflammation, or changes in energy metabolism (Alvarez‐Mora et al., 2023). Although studies have shown that chronic doses of diazepam slow down weight gain (Sonn et al., 2022), the underlying cause is not fully known. The results of the current study show that the increase in lipid metabolism may be related to this, and more targeted studies can be conducted on this subject in the future.

In the literature, significant effects of plants examined in metabolomic studies have focused on assessing protective changes in metabolic pathways caused by plant administrations after inducing pathology in animals (Cao et al., 2018). In the current study, changes in the metabolites due to direct AT exposure in the rats were examined without causing any pathology. Trehalose is an α‐diglucoside that is synthesized in most prokaryotic and eukaryotic organisms except vertebrates (Bahri et al., 2021). It did not show any toxic effects when administered to animals and was shown to induce the expression of antioxidant factors (Bahri et al., 2021). As a result of the enrichment analysis of the current study, it was determined that trehalose was significantly lower in the AT100‐administered group compared to the control (Figure 3b). Increased trehalose degradation, according to enrichment analysis, can be considered an indicator of the important physiological impact of AT100 administration in rats. In contrast, in the enrichment analysis, the AT–control group comparison results have been shown to increase the phosphatidylinositol phosphate pathway (Beziau et al., 2020) and the spermidine and spermine biosynthesis pathways, which are important for the healthy survival of the cell (Zhang et al., 2021). It can be thought that the increase in both metabolite pathways supports the protective role of AT in vivo. There is a need for further studies on the effect of AT on the sub‐pathways of these mechanisms. While the metabolomics data in the current study showed that AT increased the activity of many pathways that support cell metabolism and vital cycles, no antioxidant effects were observed in the examinations we performed in three different tissues. It has been shown in previous studies that the administration of AT extracts reverses the oxidative effect of the induced pathologies (Atila et al., 2019; Takim, 2015). However, since these studies do not provide information about the oxidant–antioxidant effect of lone AT extract without any pathology, the current study provides data for the literature.

### 4.5 Chemical composition

*Allium* is a diverse genus that is commonly used for food and medicinal purposes worldwide (Ahmed et al., 2021). *Allium* species are widely distributed in the Northern Hemisphere (Fritsch & Abbasi, 2008). In Türkiye, the diversity of *Allium* species is rich, and it holds an important place in terms of its different uses. However, there is insufficient information about its diverse chemical and biological contents.

In the literature, different analytical techniques, including high‐performance liquid chromatography and LC–MS, have been used to determine the chemical composition of *Allium* species. Among these techniques, the total phenolic content (TPC) of AT extracts was analyzed using the Folin–Ciocalteu method, and water extracts were found to have the richest TPC (Karaaslan et al., 2019; Karakavuk, 2021). On the other hand, the molecular compositions of each extract have not been evaluated due to the lack of selectivity of the Folin–Ciocalteu method. Selective methods like LC–MS analysis allow the identification of the molecular composition of the extract. Takim et al. found that quinic acid, malic acid, and vanillic acid were detected as the main phenolic acids in the methanolic extract of AT (Takim et al., 2021). The change in the solvent type emerged as a factor affecting both the TPC and the major phenolic compound (Khoddami et al., 2013; Takim, 2020). Takim et al. identified 37 phenolic compounds and found malic acid, kainic acid, cinnamic acid, vanillic acid, and fumaric acid as the major phenolic components of the AT ethanol/water extract. However, high‐pressure liquid chromatography showed only 7 phenolic compounds, with fumaric acid and catechin as the major phenolic components (Takim & Kutlu, 2020). It was observed that the number and content of phenolic compounds in the same plant extract might differ depending on the method used. Sehitoglu et al. compared the phenolic content of AT and AS methanol extracts using LC–MS/MS and found that the amount of p‐coumaric acid was significantly higher than that in AS, which may indicate a higher antioxidant capacity in AT (Rahmani et al., 2020; Şehitoglu et al., 2018).

A fatty acid analysis showed that AT has a higher amount of unsaturated fatty acids compared to AS. In contrast, its saturated fatty acid content is slightly lower, indicating that AT has a more effective basic omega acid level (Şehitoglu et al., 2018). Ugur et al. identified 15 out of 37 fatty acids in AT, primarily polyunsaturated fatty acids, including linoleic acid, and monounsaturated fatty acids, including oleic acid. Small amounts of saturated fatty acids, including palmitic acid and stearic acid, were also detected (Ugur et al., 2021). According to the GC–MS results of the current study, similar to other studies on AT (Demirci Kayiran et al., 2019), saturated fatty acids, such as palmitic acid and stearic acid, were found in the fatty acid composition.

According to Takim et al., the essential oil composition of AT consists of diallyl disulfide, diallyl trisulfide, and allyl methyl trisulfide, as well as the fatty acid composition, including oleic acid, linoleic acid, and elaidic acid, and major elements in the mineral content, such as potassium, calcium, and magnesium (Takim & Kutlu, 2020). In the current study, it was observed that medium‐chain azelate and azelaic acid, long‐chain (Z)‐9,12,13‐trihydroxyoctadec‐15‐enoic acid, and stearic acid were present in the fatty acid composition of AT, with tetracosanoic acid being the most abundant among the very long‐chain fatty acids.

The same chemical composition is not always observed in different AT species. This variation in the plants may be due to many factors, including the solvent used, the extraction method, the duration of the process, the temperature of the solvent, and the analysis method used for the extract. Even when the solvent and extraction technique are the same, the variation in the major metabolite content suggests that the season when the plant was harvested, the method and duration of drying, or biological variations between species, may be effective.

## 5 CONCLUSION

*Allium* species are known for their different biological effects, such as antioxidant, anti‐inflammatory, anxiolytic, and analgesic. The AT and diazepam groups examined in this study spent more time in the open arm, and the number of entries into the open arms was higher compared to the control group in the EPM. However, no statistical difference was observed between the groups. In this sense, it may be possible to associate the observed results with the administration period, dose, or individual characteristics of the animals included in the experiment. In particular, further studies using methanol‐prepared extracts of AT administered at longer durations and higher doses may reveal statistically significant results in terms of different behavioral parameters.

In the literature, the antioxidant and protective effects of AT administration after triggering a metabolic problem were examined. However, in the current study, short‐term administration of AT without any metabolic problems may be the reason why no significant antioxidant effect was observed. In the aforementioned studies, the antioxidant effect of AT administration on pathology‐induced groups gave significant results when compared to the lone pathology groups. However, in those studies, the antioxidant effect of lone AT administration was not mentioned in comparison with the control group. Therefore, this current study provides data for the scientific literature. It was shown in the current study that diazepam significantly decreased antioxidant levels compared to the control, which represents the negative aspect of the anxiolytic chemical in terms of oxidative stress.

The current study questioned which metabolites and pathways were augmented following the AT and diazepam exposure. In the AT100 and control group comparisons, alterations were observed in trehalose degradation, phosphatidylinositol phosphate metabolism, and spermidine biosynthesis. Between the diazepam and control groups, statistical significance was found in the beta‐oxidation of very long‐chain fatty acids, gluconeogenesis, galactose metabolism, malate aspartate shuttle, and glycine serine metabolism. We suppose that the chemical results in the current study, together with enrichment and multivariate analysis, bring a new perspective to the studies investigating the effects of *Allium* species as well as diazepam.

The phenolic components, mainly fumaric acid, malic acid, vanillic acid, quercetin‐3‐arabinoside, hydrocinnamic acid, and gallocatechin, were determined in the extract. In addition, arbutin, salicylic acid, trehalose, and nicotinic acid were analyzed in the *Allium* extract for the first time. Therefore, this study also brings new data into this field.

This study has certain limitations. In this study, no histopathology assessment was performed. The effect of *Allium* extracts on tissues such as the liver or kidney can be conducted in further studies. In addition, high doses and extracts obtained with different solvents, such as methanol, were not used and compared as in the literature. Literature studies have examined the protective effects of *Allium* in terms of antioxidants or other parameters after inducing pathologies. Although the current study includes different aspects such as metabolomics, antioxidant activity, and behavioral studies, only healthy animals were used.

The promising potential of *Allium* species, as given in the literature, in the discovery of anxiolytic compounds that do not cause oxidative stress in mammalian tissues needs attention. Further in vivo studies in this field can reveal the detailed impacts of AT and other *Allium* species, since they have an important place in the human diet and also in ethnobotany.

## AUTHOR CONTRIBUTIONS

**Nigar Abbak:** Conceptualization (equal); formal analysis (lead); investigation (lead); resources (equal); writing – original draft (lead); writing – review and editing (lead). **Emirhan Nemutlu:** Investigation (equal); methodology (equal); resources (equal); validation (equal); writing – original draft (equal); writing – review and editing (equal). **Tuba Reçber:** Investigation (supporting); methodology (supporting); validation (supporting). **Asli San Dagli Gul:** Conceptualization (equal); formal analysis (supporting); investigation (supporting); writing – original draft (equal). **H. Turan Akkoyun:** Investigation (equal); methodology (equal); writing – original draft (equal). **Mahire Bayramoglu Akkoyun:** Investigation (equal); methodology (equal); writing – original draft (equal). **Gulderen Yilmaz:** Investigation (supporting); methodology (supporting); validation (supporting). **Suat Ekin:** Methodology (supporting); validation (supporting). **Ahmet Bakir:** Methodology (supporting); validation (supporting). **Okan Arihan:** Conceptualization (equal); formal analysis (equal); investigation (equal); methodology (equal); writing – original draft (equal); writing – review and editing (equal).

## ACKNOWLEDGEMENTS

We would like to thank Hacettepe University Faculty of Medicine, Department of Physiology and Faculty of Pharmacy, Department of Analytical Chemistry for allowing us to work in their laboratories.

## FUNDING INFORMATION

No funding was received for this study.

## CONFLICT OF INTEREST STATEMENT

The authors declare no conflicts of interest.

## ETHICAL APPROVAL

The study was conducted after getting ethical approval from the Hacettepe University Animal Experiments Ethical Committee (approval no. 2022/10–04). All of the experiments were conducted by following the National Institute of Health Guide for the Care and Use of Laboratory Animals.

## CONSENT FOR PUBLICATION

All of the authors agreed to the publication of this manuscript.

## Data Availability

Research data are not shared.
